# Evaluation of Social Media Short‐Form Video Content for Patient Education on Vision‐Threatening Diseases

**DOI:** 10.1155/joph/8987000

**Published:** 2026-02-18

**Authors:** Riya H. Patel, David Mothy, Neha Boinpally, Hassaam S. Choudhry, Puja Bhavsar, Albert S. Khouri

**Affiliations:** ^1^ Department of Ophthalmology and Visual Science, Rutgers New Jersey Medical School, Newark, New Jersey, USA, rutgers.edu; ^2^ Department of Biological Sciences, New Jersey Institute of Technology, Newark, New Jersey, USA, njit.edu

## Abstract

**Purpose:**

The rapid spread of unreliable misinformation on vision‐threatening diseases can significantly affect the eye health behaviors and outcomes of the patients consuming short‐form social media content. This study evaluates short‐form videos pertaining to vision‐threatening diseases to quantify video quality, content, and popularity.

**Methods:**

This cross‐sectional study analyzed short‐form videos on cataracts, diabetic retinopathy, glaucoma, and age‐related macular degeneration from TikTok, Instagram Reels, and YouTube Shorts. A hashtag search identified the first fifty videos on each disease from each social media platform. Two reviewers evaluated them, resolving discrepancies with a third. Outcome measures included number of views, likes, comments, uploader source, content type, modified DISCERN score (0–5 scale), and global quality scale (GQS) score (1–5 scale). Engagement outcomes were summarized descriptively using medians and interquartile ranges, while reliability and quality outcomes were analyzed using one‐way ANOVA with Tukey post hoc comparisons.

**Results:**

TikTok videos demonstrated higher median engagement (views, likes, and comments) compared to Instagram Reels and YouTube Shorts. Videos on cataracts had higher engagement statistics compared to the other vision‐threatening diseases across all platforms. Physicians were the most common video source (45%). The most common content categories were treatments/management (36%) and general symptoms (22%). YouTube Shorts had a significantly greater average DISCERN (2.93 ± 0.70) and GQS score (3.85 ± 1.26) than Instagram Reels and TikTok (*p* < 0.001). Videos from patients had the lowest mean DISCERN and GQS scores.

**Conclusions:**

TikTok had the greatest median engagement levels, while YouTube Shorts had the greatest mean quality and reliability. Videos from patients and philanthropists had lower quality scores, while healthcare professionals and organizations had the highest. Future efforts should understand the patients’ perspectives, address misinformation, and improve quality across all social media platforms.

## 1. Introduction

Vision‐threatening eye diseases are the major cause of visual impairment and blindness worldwide [1]. Specifically, cataracts, age‐related macular degeneration (AMD), glaucoma, and diabetic retinopathy (DR) are the four major vision‐threatening diseases that account for increasing incidences of vision loss. According to the American Academy of Ophthalmology, in the United States, 20.5 million people have cataracts, 9.6 million people have DR, 3 million people have glaucoma, and 1.5 million people have AMD [2]. The global burden of vision loss and blindness is increasing exponentially due to an aging population, urbanization, and increasing levels of screen time [3]. It is important to understand the prevention, management, and treatment of these eye conditions to improve the quality of life of patients, starting with proper patient education.

The Internet and social media platforms have accelerated instantaneous information sharing in the past decade. In healthcare, social media can be a valuable tool by allowing patients to share their experiences as well as seek education, advice, and information regarding their health [4, 5]. Recently, short video clips, such as TikTok, have increased in popularity [6, 7]. These short video clips often range from 5 to 60 s, providing quick bits of information and content to viewers. However, social media platforms and the information they disseminate are unregulated and thus can be misleading, inaccurate, or harmful to patients if not used properly. Furthermore, over 50% of healthcare professionals recommend that patients use online education resources, but only a few of these professionals actually express confidence in the reliability and quality of these resources [8].

A recent study by Sampige et al. discovered that ophthalmology content on TikTok often contained misinformation [9]. Other studies on ophthalmology content on TikTok demonstrated that there were a limited number of videos created by ophthalmologists and other eye care providers, possibly indicating poor reliability of information [10, 11]. Similar findings of poor video quality and misleading content on social media platforms have also been observed in other specialties of medicine such as dermatology [12–15], plastic surgery [16], otolaryngology [17, 18], and orthopedic surgery [5, 19]. However, all these studies on short‐form videos only evaluate TikTok, even though there has been a recent rise of other social media platforms also adopting short‐form video content. These platforms include Instagram Reels and YouTube Shorts, which are increasing in popularity as alternatives to TikTok and also require evaluation of healthcare content.

Misinformation, especially on conditions as severe as vision‐threatening diseases, has implications on altering patient perceptions and education. Since it has been shown that patients often turn to social media for medical information and that social media content alters health‐related behaviors, poor‐quality short‐form videos on vision‐threatening diseases are a cause for concern that can misguide patients in the screening, diagnosis, and management of these conditions [20, 21]. Thus, it is crucial to evaluate the quality, informational content, and engagement statistics of short‐form videos on vision‐threatening diseases across multiple social media platforms to ensure that patients are consuming accurate and reliable information. The purpose of this study is to evaluate videos pertaining to the four vision‐threatening diseases (cataracts, DR, glaucoma, and AMD) on TikTok, Instagram Reels, and YouTube Shorts to quantify video quality, content, and popularity.

## 2. Methods

On March 14, 2024, short‐form videos on the four vision‐threatening diseases were searched for on TikTok, Instagram Reels, and YouTube Shorts using the following hashtags: “cataracts,” “diabetic retinopathy,” “glaucoma,” and “age‐related macular degeneration.” The first 50 videos on each social media platform on each disease that fit within criteria were included, resulting in 600 videos analyzed in this study. We selected the first 50 videos in the order they appeared when we searched for the disease, as we aimed to replicate the health information a typical patient would see in their search. Inclusion criteria were videos on TikTok, Instagram Reels, and YouTube Shorts pertaining to cataracts, DR, glaucoma, and AMD. Exclusion criteria were videos that were repeated, not in English, irrelevant, or lacking audio [18]. All the data that were collected were from publicly available sources, and no human subjects or personally identifiable data were analyzed. Institutional review board approval was not required.

For the 600 short‐form videos, metadata on engagement data (including title, video duration, number of views/likes/comments, video source/uploader, type of content, posting date, and URL link) was recorded. Video sources were classified into the categories of academic (affiliated with research groups, universities, or colleges), physician (independent physician or physician group without research, university, or college affiliation), nonphysician health professional, medical source, patient, commercial, philanthropic, or other. Types of content were classified into the categories of general symptoms, disease pathophysiology, symptoms, treatments/management, patient experience, procedural video, or other. All videos were then evaluated for reliability using the validated modified DISCERN tool and for quality using the global quality scale (GQS) [4, 5, 22, 23].

The modified DISCERN scale consists of five adapted questions from the 16 questions from the original DISCERN scale as seen in Table 1. For each of the five questions, a value of 1 indicates a “yes” and a value of 0 indicates a “no.” These points are summed to result in a score from 0 (low reliability) to 5 (high reliability). The GQS consists of five descriptions regarding the quality, flow of information, and utility for patients on a scale of 1 (poor quality) to 5 (high quality) as seen in Table 2. Each video was independently evaluated by two reviewers, and any discrepancies in ratings were resolved independently by a third reviewer. All three reviewers were medical trainees with prior research experience in ophthalmology and received standardized training on using the modified DISCERN and GQS scoring tools to ensure inter‐rater consistency and reliability. Engagement metrics (views, likes, and comments) demonstrated substantial right skew and were therefore summarized descriptively using medians and interquartile ranges (IQRs), without inferential hypothesis testing. Reliability and quality outcomes (modified DISCERN and GQS scores) were analyzed using one‐way ANOVA with Tukey post hoc comparisons. Categorical variables were analyzed using chi‐squared tests. A *p* value less than or equal to 0.05 was considered statistically significant. Data management and production of figures were conducted on Microsoft Excel, Version 16.98. Statistical analyses were performed using IBM SPSS Statistics for Windows, Version 31.0.

**TABLE 1:** The modified DISCERN tool (1 point for every yes, 0 point for every no).

Score	Question
1	Are the aims clear and achieved?
2	Are reliable sources of information used?
3	Is the information presented balanced and unbiased?
4	Are additional sources of information listed for patient reference?
5	Are areas of uncertainty motioned?

**TABLE 2:** Global quality score (scored based on the following characteristics).

Score	Question
1	Poor quality, poor flow, most information missing, not useful for patients.
2	Generally poor, some information given but of limited use to patients.
3	Moderate quality, some important information is adequately discussed.
4	Good quality, good flow, most relevant information is covered, useful for patients
5	Excellent quality and excellent flow, very useful for patients.

## 3. Results

A total of 600 short‐form videos were included in this study. For each of the four vision‐threatening diseases, 50 videos on each of the three social media platforms were evaluated for engagement, reliability, and quality. Average engagement statistics, classification of content, and average DISCERN and GQS scores across TikTok, Instagram Reels, and YouTube Shorts are included in Table 3. The most common uploader source was physicians, with most videos having a focus on treatment/management. The 600 videos scored an average of 2.55 ± 0.97 on the modified DISCERN tool for reliability and a 3.35 ± 1.33 on the GQS tool for quality.

**TABLE 3:** Summary of short‐form videos on vision‐threatening diseases from all platforms.

**Engagement**	**Median**	**IQR**

Duration (seconds)	40	21–59
Views	4475	693–35800
Likes	107	18–738
Comments	3	0–26

**Uploader sources**	**(%)**	

Academic	4	
Physician	45	
Nonphysician	11	
Medical source	20	
Patient	9	
Commercial	8	
Philanthropic	2	
Other	1	

**Type of content**	**(%)**	

General symptoms	21	
Disease pathophysiology	17	
Symptoms	1	
Treatments/management	36	
Patient experience	19	
Procedural video	5	
Other	3	

**Evaluation**	**Mean**	**Standard deviation**

DISCERN Score	2.55	0.97
Global quality score (GQS)	3.35	1.33

TikTok had greater engagement statistics compared to Instagram Reels and YouTube Shorts (Supporting Table 1). TikTok had a higher median number of views of 29,000 (IQR: 4564–149050) compared to Instagram Reels with a median number of views of 6679 (IQR: 1408–24540) and YouTube Shorts with a median number of views of 606 (IQR: 171–2411). TikTok had a median of 496 (IQR: 93–3256) likes per video, whereas Instagram Reels only had a median of 168 (IQR: 43–813) likes per video and YouTube Shorts only had a median of 17 (IQR: 4–64) likes per video. Similarly, TikTok had the greatest median number of comments per video of 27 (IQR: 4–85) compared to Instagram Reels with a median number of comments per video of 5 (IQR: 1–18) and YouTube Shorts with a median number of comments per video of 0 (IQR: 0‐1). Engagement statistics of the four vision‐threatening diseases within each social media platform were also evaluated and are summarized in Supporting Table 1.

**FIGURE 1:**
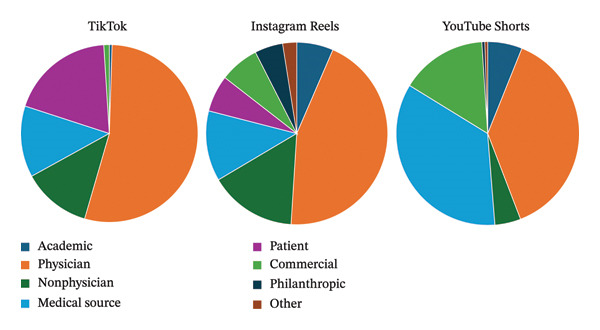
Short‐form videos categorized based on social media platform and video uploader source.

**FIGURE 2:**
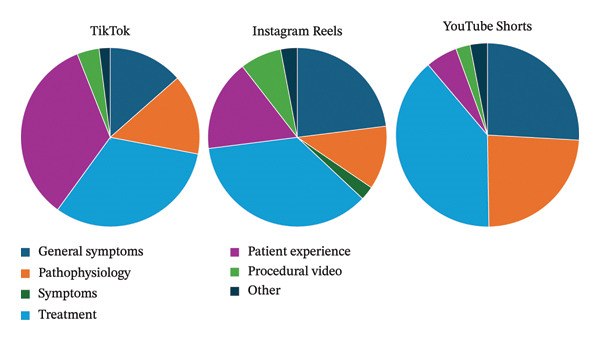
Short‐form videos categorized based on social media platform and content type.

Across all three platforms, physicians were the most common uploader source (Figure 1). After physicians, patients were the most common uploaders on TikTok, nonphysician health professionals were most likely to post on Instagram Reels, and medical sources (i.e., health websites) were most likely to post on YouTube Shorts. On Instagram Reels and YouTube Shorts, treatments and management were the most common type of content (Figure 2). However, on TikTok, patient experience videos were the most common type of content.

**FIGURE 3:**
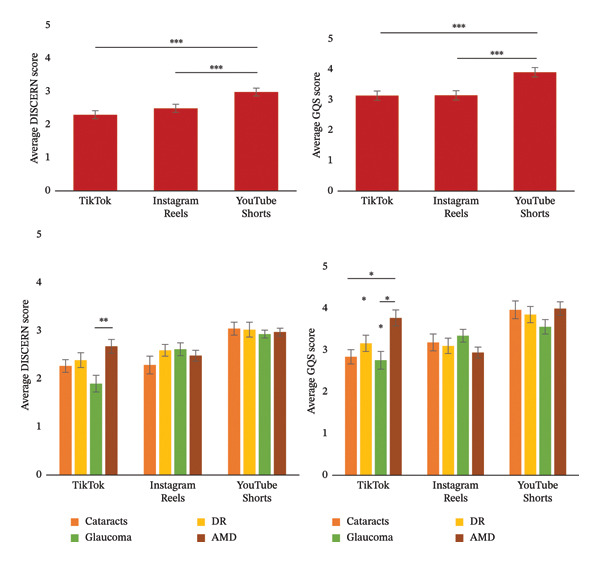
Reliability and quality of short‐form videos as measured by average DISCERN and GQS scores. ^∗∗^denotes significance at *p* < 0.01; ^∗∗∗^denotes significance at *p* < 0.001.

Statistically significant results were found when comparing the reliability and quality of these short‐form videos on different media platforms, as measured by the modified DISCERN score and GQS score, respectively (Figure 3). YouTube Shorts had the greatest modified DISCERN score (2.93 ± 0.70), compared to TikTok (2.26 ± 1.07; *p* < 0.001) and Instagram Reels (2.45 ± 0.97; *p* < 0.001). YouTube Shorts also had the greatest GQS score (3.85 ± 1.26) compared to TikTok (3.09 ± 1.44; *p* < 0.001) and Instagram Reels (3.10 ± 1.12; *p* < 0.001). When comparing the difference in reliability and quality of short‐form videos between each of the four vision‐threatening diseases, statistical differences were only found between the videos on TikTok. Videos focusing on AMD had a higher modified DISCERN score (*p* = 0.002) and GQS score (*p* = 0.002) compared to videos focusing on glaucoma. Additionally, videos on AMD had a higher GQS score compared to videos on cataracts (*p* = 0.006).

**FIGURE 4:**
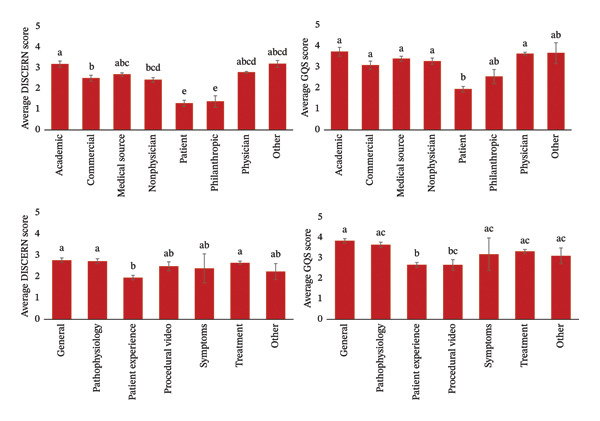
Reliability and quality of short‐form videos as measured by average DISCERN and GQS scores based on uploader source and content type. Different letters between two bars indicate a statistically significant difference of *p* < 0.001. Results are further summarized in Supporting Tables 1 and 2.

The reliability and quality of these short‐form videos demonstrated significant differences based on the uploader source (Figure 4). Most notably, the average DISCERN scores for short‐form videos uploaded by patients (1.27 ± 1.06) and philanthropic organizations (1.36 ± 0.92) were significantly lower than the average DISCERN scores for short‐form videos uploaded by other sources (*p* < 0.001). The uploaders with the highest average DISCERN scores were academic institutions (3.15 ± 0.78), medical sources (2.66 ± 0.86), and physicians (2.76 ± 0.80; *p* < 0.001). Similar trends are seen when comparing the average GQS scores of short‐form videos uploaded by different sources. The average GQS scores for short‐form videos uploaded by patients (1.94 ± 0.96) and philanthropic organizations (2.55 ± 1.13) were significantly lower than the average GQS scores for short‐form videos uploaded by other sources (*p* < 0.001). When comparing short‐form videos based on content type, patient experience videos had a lower average DISCERN score (1.96 ± 1.11) and GQS score (2.68 ± 1.19) compared to other content types, such as disease pathophysiology and treatments/management (*p* < 0.001). Procedural videos also had a lower average GQS score (2.68 ± 1.36) when compared to other short‐form video content types (*p* < 0.001).

## 4. Discussion

Our evaluation of 600 short‐form videos on four different vision‐threatening diseases (cataracts, DR, glaucoma, and AMD) across three social media platforms (TikTok, Instagram Reels, and YouTube Shorts) is the first study to understand the popularity, quality, and reliability of ophthalmology content across different platforms and pathologies. With the increasing popularity of short‐form content on social media, the risk of the rapid spread of misinformation and widespread poor patient education also increases [6]. This is specifically concerning in ophthalmology since poor understanding about severe pathologies, such as vision‐threatening diseases, can lead to irreversible blindness and vision loss. It is crucial to evaluate the short‐form videos that the general population is consuming to avoid patients receiving inaccurate information or biased perspectives on vision‐threatening conditions. Since TikTok content has been evaluated in previous literature, out inclusion of Instagram Reels and YouTube Shorts highlights the importance of evaluating a variety of media sources to capture differences in the ophthalmology content.

We demonstrated there are significant discrepancies between the popularity and quality/reliability of short‐form videos on vision‐threatening diseases. For example, short‐form videos uploaded on TikTok had higher popularity and engagement (as measured by median number of views, likes, and comments) compared to YouTube Shorts and Instagram Reels. However, these videos on TikTok had lower reliability and quality (as measured by average DISCERN and GQS scores) compared to YouTube Shorts and Instagram Reels. This demonstrates that there is the greatest level of engagement with the lowest quality videos on vision‐threatening diseases, indicating that many viewers may be consuming videos with misinformation or unreliable content. Although Instagram Reels and YouTube Shorts are catching up to TikTok in terms of usage and popularity, it is crucial to maximize better patient education by ensuring that the highest quality videos are being consumed by the largest audience.

Of the four vision‐threatening diseases we evaluated in this study, videos on cataracts seemed to have the greatest popularity on TikTok, Instagram Reels, and YouTube Shorts. It is interesting to note that the engagement of cataract content, which is a reversible vision‐threatening disease, is higher compared to DR, glaucoma, and AMD, which are typically more detrimental and irreversible. This demonstrates that patients are consuming less engaging content on these more complex conditions, which is a concern in the management and outcomes of patients with DR, glaucoma, and AMD using social media. Although there were no major significant differences in the reliability and quality of short‐form videos between the diseases, videos on AMD did have higher DISCERN and GQS scores on TikTok. However, the overall quality, reliability, and engagement of short‐form videos on vision‐threatening diseases were poor and inconsistent across different social media platforms and require significant improvement for patient education.

More inconsistencies were identified when looking at the reliability and quality of short‐form videos based on uploader source and content type. Specifically, videos uploaded by patients or about the patient experience had lower quality and reliability scores when compared to other uploader sources (such as physicians, academic institutions, and other medical sources) and content types (such as disease pathophysiology and treatment/management). This may be due to the inherently subjective patient experience and lack of formal education on vision‐threatening diseases by patients. On the other hand, patients can provide valuable first‐hand information on vision‐threatening diseases that can improve awareness and advocacy of ophthalmic conditions. Previous studies have also reported similar findings in which ophthalmology social media videos uploaded by laypeople had lower DISCERN scores compared to medical professionals [11, 18]. It is crucial to balance the benefit of sharing the patient experience and poor quality and reliability of patient videos on short‐form social media platforms. Videos uploaded by philanthropic organizations also had poorer quality and reliability scores. Other nonhealthcare‐related video uploader sources, such as commercials, also consist of a considerable portion of video uploaders. Users should pay extra attention to the videos they consume that are uploaded by the groups that are not validated healthcare experts to evaluate the true quality and reliability of the information they are receiving. As previously mentioned, physicians, academic institutions, and other medical sources uploaded the highest quality and reliable short‐form videos on vision‐threatening diseases. The improved quality and reliability of videos uploaded by medical professionals are also demonstrated in other studies looking at ophthalmology content on social media [9–11, 24] as well as in other medical specialties such as otolaryngology [18], dermatology [12, 13, 25], and plastic surgery [16]. This emphasizes the need for physicians and other trained and reputable medical sources to increase their presence on various social media platforms to create high‐quality content for patient education.

Our study does have some limitations. Although a total of 600 short‐form videos were evaluated, only 50 videos per vision‐threatening disease on each social media platform were analyzed. Though we chose to evaluate the first 50 English videos that appeared based on our searches, they still only represent a small proportion of all short‐form videos on ophthalmology that users may consume. Additionally, our goal was to stimulate the user experience in viewing short‐form videos on vision‐threatening diseases, but since cross‐sectional studies are limited by evaluating data at a single point‐in‐time, our methodology is not fully consistent with long‐term social media user experiences. Due to the dynamic nature of social media and new videos constantly being uploaded, it is difficult to truly evaluate the full breadth of ophthalmology content on social media platforms at different points in time [5]. This study was designed as an exploratory, descriptive analysis aimed at comparing overall patterns on social media. Engagement metrics were analyzed descriptively due to substantial right skew, which precluded valid parametric inference. Medians and IQRs were therefore reported to provide a more representative summary of observed engagement patterns. Finally, characterizing the misinformation found in these short‐form videos was not completed in this study but could be helpful to further understand and correct the specific problems associated with videos shared on social media [9]. Despite these limitations, our evaluation of short‐form content across multiple social media platforms provides a strong foundational understanding of the engagement, quality, and reliability of major vision‐threatening diseases in ophthalmology.

As medical‐related content continues to grow on social media, future efforts should focus on ensuring that high‐quality and reliable content is prioritized and promoted for engagement by social media users to avoid misinformation and improve patient education. Specifically, increasing the quality and reliability of videos on TikTok as well as increasing the engagement of videos on YouTube Shorts can help close the gap between social platforms, regarding the poorest quality videos being consumed by the largest audience. Moreover, physicians, academic institutions, and other reputable medical sources should continue to upload high‐quality and reliable content on TikTok, Instagram Reels, and YouTube Shorts as they have done in the past. Finally, social media platforms can adopt methods for fact‐checking, which is currently unavailable, to limit posts that contain misinformation and boost posts that contain high‐quality, accurate information from reliable sources. This screening of social media content can ensure that patients are consuming the highest quality medical content and are not misguided when making significant medical decisions.

Future studies can focus on the reliability and content of long‐form videos such as YouTube videos and comparing them to short‐form videos. Furthermore, another future study can explore ophthalmologists’ opinions on these short‐form videos to better understand the extent to which these videos serve as an educational resource for others. Regarding the gravity of vision‐threatening diseases, these efforts can decrease the consequences of undiagnosed ophthalmic conditions and incidences of avoidable vision loss in patients.

## 5. Conclusion

Our analysis of short‐form videos on four different vision‐threatening diseases across three popular social media platforms aims to understand patients’ social media consumption of ophthalmology content. Significant differences in the popularity, quality, and reliability of short‐form videos were observed across different platforms, pathologies, video uploaders, and video content types. Harnessing social media content, specifically short‐form videos, as a tool for improving patient education and minimizing misinformation on vision‐threatening diseases can revolutionize ophthalmic care and improve patient quality of life.

## Funding

The authors declare that no funds, grants, or other support was received for this research.

## Conflicts of Interest

Albert S Khouri M.D. reports grants from NJ Health Foundation and Fund for the NJ Blind as well as consulting and lecture fees from Glaukos, Alcon, Bausch and Lomb, and AbbVie. All other authors declare no conflicts of interest.

## Supporting Information

Additional supporting information can be found online in the Supporting Information section.

## Supporting information


**Supporting Information1** Supporting Table 1. Analysis of short‐form videos on vision‐threatening diseases on TikTok vs. Instagram Reels vs. YouTube Shorts.

**Supporting Information2** Supporting Table 2. Analysis of short‐form videos on vision‐threatening diseases based on video uploader and video content.

## Data Availability

The data that support the findings of this study are available from the corresponding author upon reasonable request.
