# Supplementary material for: Evaluation of Social Media Short‐Form Video Content for Patient Education on Vision‐Threatening Diseases
Source: J Ophthalmol. 2026 Feb 18;2026:8987000. doi: 10.1155/joph/8987000 (PMC12917257; doi:10.1155/joph/8987000)
Supplement: Supplementary file 1 — Supporting Information1 Supporting Table 1. Analysis of short‐form videos on vision‐threatening diseases on TikTok vs. Instagram Reels vs. YouTube Shorts. [file JOPH-2026-8987000-s002.docx]

***Supplementary Table 1. Analysis of Short-Form Videos on Vision Threatening Diseases on TikTok vs. Instagram Reels vs. YouTube Shorts***

|  |  | **TikTok** | **Instagram Reels** | **YouTube Shorts** |
| --- | --- | --- | --- | --- |
| Engagement - All Vision Threatening Diseases | | | | |
|  | Median Views (IQR) | 29000 (4564-149050) | 6679 (1408-24540) | 606 (171-2411) |
|  | Median Likes (IQR) | 96 (93-3256) | 168 (43-813) | 17 (4-64) |
|  | Median Comments (IQR) | 27 (4-85) | 5 (1-18) | 0 (0-1) |
| Engagement - Cataracts | | | | |
|  | Median Views (IQR) | 111000 (33050-648450) | 16116 (5624-77272) | 1123 (177-3625) |
|  | Median Likes (IQR) | 1461 (395-10253) | 375 (163-2110) | 24 (4-139) |
|  | Median Comments (IQR) | 56 (26-170) | 11 (4-53) | 0 (0-3) |
| Engagement - Diabetic Retinopathy | | | | |
|  | Median Views (IQR) | 24800 (5736-154725) | 7049 (1858-24321) | 484 (145-1521) |
|  | Median Likes (IQR) | 339 (109-1812) | 173 (43-698) | 10 (5-61) |
|  | Median Comments (IQR) | 26 (4-104) | 6 (1-15) | 0 (0-2) |
| Engagement - Glaucoma | | | | |
|  | Median Views (IQR) | 57900 (19750-219400) | 2278 (1036-10749) | 1138 (334-3767) |
|  | Median Likes (IQR) | 1581 (313-6850) | 63 (26-207) | 23 (7-71) |
|  | Median Comments (IQR) | 54 (13-127) | 2 (0-5) | 0 (0-2) |
| Engagement - Age Related Macular Degeneration | | | | |
|  | Median Views (IQR) | 1969 (677-12625) | 3550 (575-17172) | 287 (104-767) |
|  | Median Likes (IQR) | 40 (15-284) | 155 (27-792) | 9 (3-25) |
|  | Median Comments (IQR) | 2 (0-15) | 3 (0-14) | 0 (0-25) |
| Uploader Sources | |  |  |  |
|  | Academic | 0.5 | 6.5 | 6 |
|  | Physician | 54 | 44.5 | 37.5 |
|  | Non-physician | 12.5 | 15.5 | 4.5 |
|  | Medical source | 13 | 12.5 | 34.5 |
|  | Patient | 19 | 6.5 | 0 |
|  | Commercial | 1 | 7 | 15 |
|  | Philanthropic | 0 | 5 | 0.5 |
|  | Other | 0 | 2.5 | 0.5 |
| Type of Content | |  |  |  |
|  | General symptoms | 13.5 | 23 | 25.5 |
|  | Pathophysiology | 14.5 | 11.5 | 23.5 |
|  | Symptoms | 0 | 2.5 | 0 |
|  | Treatment | 32 | 36 | 38.5 |
|  | Patient experience | 34 | 16.5 | 5.5 |
|  | Procedural Video | 4 | 7.5 | 2.5 |
|  | Other | 2 | 3 | 3 |
| Evaluation - All Vision Threatening Diseases | | | | |
|  | Average DISCERN Score ± SD | 2.26 ± 1.07 | 2.45 ± 0.97 | 2.93 ± 0.70 |
|  | Average Global Quality Score ± SD | 3.09 ± 1.44 | 3.1 ± 1.12 | 3.85 ± 1.26 |
| Evaluation - Cataracts | |  |  |  |
|  | Average DISCERN Score ± SD | 2.22 ± 0.95 | 2.24 ± 1.27 | 2.98 ± 0.89 |
|  | Average Global Quality Score ± SD | 2.80 ± 1.40 | 3.14 ± 1.20 | 3.96 ± 1.46 |
| Evaluation - Diabetic Retinopathy | |  |  |  |
|  | Average DISCERN Score ± SD | 2.34 ± 1.06 | 2.54 ± 0.86 | 2.94 ± 0.74 |
|  | Average Global Quality Score ± SD | 3.12 ± 1.36 | 3.06 ± 1.25 | 3.88 ± 1.25 |
| Evaluation - Glaucoma | |  |  |  |
|  | Average DISCERN Score ± SD | 1.86 ± 1.18 | 2.56 ± 0.88 | 2.90 ± 0.58 |
|  | Average Global Quality Score ± SD | 2.72 ± 1.51 | 3.30 ± 1.09 | 3.62 ± 1.21 |
| Evaluation - Age Related Macular Degeneration | | | | |
|  | Average DISCERN Score ± SD | 2.62 ± 0.97 | 2.46 ± 0.79 | 2.92 ± 0.57 |
|  | Average Global Quality Score ± SD | 3.72 ± 1.31 | 2.90 ± 0.91 | 3.96 ± 1.12 |
